# Life-Style and Genome Structure of Marine *Pseudoalteromonas* Siphovirus B8b Isolated from the Northwestern Mediterranean Sea

**DOI:** 10.1371/journal.pone.0114829

**Published:** 2015-01-14

**Authors:** Elena Lara, Karin Holmfeldt, Natalie Solonenko, Elisabet Laia Sà, J. Cesar Ignacio-Espinoza, Francisco M. Cornejo-Castillo, Nathan C. Verberkmoes, Dolors Vaqué, Matthew B. Sullivan, Silvia G. Acinas

**Affiliations:** 1 Department of Marine Biology and Oceanography, Institut de Ciències del Mar (CSIC), Passeig Marítim de la Barceloneta, 37–49, 08003 Barcelona, Spain; 2 University of Arizona, Department of Ecology and Evolutionary Biology, 1007 E. Lowell St., Tucson, AZ, United States of America; 3 University of Arizona, Department of Molecular and Cellular Biology, 1007 E. Lowell St., Tucson, AZ, United States of America; 4 Chemical Science Division, Oak Ridge National Laboratory, Oak Ridge, TN 37831, United States of America; U.S. Geological Survey, UNITED STATES

## Abstract

Marine viruses (phages) alter bacterial diversity and evolution with impacts on marine biogeochemical cycles, and yet few well-developed model systems limit opportunities for hypothesis testing. Here we isolate phage B8b from the Mediterranean Sea using *Pseudoalteromonas* sp. QC-44 as a host and characterize it using myriad techniques. Morphologically, phage B8b was classified as a member of the *Siphoviridae* family. One-step growth analyses showed that this siphovirus had a latent period of 70 min and released 172 new viral particles per cell. Host range analysis against 89 bacterial host strains revealed that phage B8b infected 3 *Pseudoalteromonas* strains (52 tested, >99.9% 16S rRNA gene nucleotide identity) and 1 non-*Pseudoaltermonas* strain belonging to *Alteromonas* sp. (37 strains from 6 genera tested), which helps bound the phylogenetic distance possible in a phage-mediated horizontal gene transfer event. The *Pseudoalteromonas* phage B8b genome size was 42.7 kb, with clear structural and replication modules where the former were delineated leveraging identification of 16 structural genes by virion structural proteomics, only 4 of which had any similarity to known structural proteins. In nature, this phage was common in coastal marine environments in both photic and aphotic layers (found in 26.5% of available viral metagenomes), but not abundant in any sample (average per sample abundance was 0.65% of the reads). Together these data improve our understanding of siphoviruses in nature, and provide foundational information for a new ‘rare virosphere’ phage–host model system.

## Introduction

In marine environments, phages influence global biogeochemical cycles by lysing bacterial cells which alters nutrient and organic matter fluxes, as well as the dynamics and diversity of microbial communities [1–9]. Additionally, marine phages help drive microbial evolution through phage-mediated gene transfer [10]. Despite their relevance, viral diversity is hard to measure because (i) viruses lack a universally conserved gene marker (e.g., ribosomal RNA genes in cellular organisms) [11], and (ii) most (>99%) bacteria in nature are resistant to cultivation using standard techniques [12] which limits the hosts available for virus isolation efforts [13]. Even when it is possible to grow the host organism in the lab, not all phages produce identifiable plaques [6, 14]. To circumvent these limitations, viral community diversity has been analyzed by culture-independent approaches including (i) Pulse-Field Gel Electrophoresis (PFGE) which discriminates viruses by genome size [15, 16], (ii) Randomly Amplified Polymorphic DNA PCR (RAPD) which provides a genetic fingerprint for the whole viral community [17–19] and most recently (iii) viral metagenomics (viromics) which, currently, provides fragmented sequence data for the whole double-stranded DNA (dsDNA) viral community [20–28].

Culture-independent and metagenomic methods are powerful, but each approach has its own limitations. Although PFGE is often used to estimate the size of individual phage genomes, it does not efficiently discriminate among natural viral populations with the same or similar genome size. RAPD-PCR is a valid alternative although it may under- or overestimate viral richness if genetically different DNA templates produce PCR amplicons of the same size or if a single viral genome contains more than one priming site resulting in multiple bands from the same virus in the final banding pattern. Finally, metagenomic approaches are severely database limited due to the lack of sequenced viral genomes. For example, the majority (>70%) of the predicted viral open reading frames (ORFs) in metagenomes have no similarity to previously described sequences [20, 21, 26, 29, 30]. While informatic advances are eliminating some of these issues (e.g., protein cluster organization [26] and kmer-based ecological modeling [27]), the viral metagenomes themselves, while now quantitative for dsDNA viruses [31–36] are currently not capturing RNA viruses, ssDNA viruses, and giant viruses [37–41]. Thus, new methods are needed to capture RNA and ssDNA viral sequence space, and relevant and representative isolate-based genomes are essential to better map dsDNA viral sequence space and virus–host interactions in nature.

Most sequenced marine phage genomes belong to cyanophages [42], with recent addition of phages infecting other ecologically important marine heterotrophic bacteria [13, 43, 44]. Within heterotrophic bacteria, *Pseudoalteromonas* sp. strains are members of Gammaproteobacteria, and this class of Proteobacteria may comprise up to 30% of total marine bacterioplankton with a 20 to 80% of them taking up ^3^H‐leucine [45], reflecting active members of the microbial loop. Moreover, it has been shown that members of the Gammaproteobacteria bacterial group show the highest growth rates among their oceanic counterparts and they are subjected to higher viral pressure than other groups in the NW Mediterranean Sea [46]. Also, *Pseudoalteromonas* sp. are in many cases associated to particle attached bacterial assemblages, wherein it has been shown that Gammaproteobacteria abundance reached maximum peaks in the range of 24 to 60% of the total OTUs studied using pyrosequencing [47]. Several studies have reported the ecological and evolutionary importance of the *Pseudoalteromonas* phages [48–51]. For instance, the ecogenomic analysis of the marine *Pseudoalteromonas* phage H105/1 revealed the presence of several genes in estuarine samples and this phage showed evolutionary relationships with its host in some proteins and functional modules [49]. Moreover, some of the sequenced *Pseudoalteromonas* bacterial genomes showed genes indicative of integrated prophages [49, 52, 53]. However, there are only four marine *Pseudoalteromonas* phage genomes sequences available in public datasets: *Pseudoaltermonas* phage PM2 (*Corticoviridae*), *Pseudoalteromonas* phage H105/1 (*Siphoviridae*), *Pseudoalteromonas* phage RIO-1 (*Podoviridae*) and *Pseudoalteromonas* phage pYD6-A (*Podoviridae*) [49, 54, 55]. Despite infecting bacteria of the same genus, these phages do not share any genes and even belong to different phage families. While PM2 is the only sequenced corticovirus, H105/1 has functional organization similar to λ-like siphoviruses [49] and RIO-1 and pYD6-A are distantly related to T7-like and N4-like podoviruses, respectively [55]. This suggests high phage diversity and the importance of phage pressure in this bacterial group.

Thus, to better expand our understanding of *Pseudoalteromonas* phage diversity, phage–host interactions in the marine environment and the genomic features of marine phages, we isolate and characterize the *Pseudoalteromonas* phage B8b.

## Material and Methods

### Phage isolation

*Pseudoalteromonas* phage B8b was obtained from Blanes Bay Microbial Observatory (BBMO, 41° 40’ 13.5’’N 2° 48’ 00.6’’E http://www.icm.csic.es/bio/projects/icmicrobis/bbmo), a surface coastal site in the NW Mediterranean Sea, in winter 2009. No specific permissions to sample were required for this location. Four liters of surface seawater was collected and after a 0.22 μm prefiltration (Millipore, Whatmann), phages were concentrated by tangential flow filtration (30KDa VIVAFLOW cartridge, Sartorius) to a final volume of 20 ml. Phages were isolated using liquid enrichment cultures and plaque assays [56]. The host strain was *Pseudoalteromonas* sp. QC-44 (accession number: KM609273); which was isolated from the same marine site in 2009 using Zobell medium (1.0 g yeast extract, 5 g peptone, 15 g agar and 250 ml MQ water and 750 ml 30 kDa filtered seawater). This isolate was chosen because it was highly retrieved from our marine site. In the enrichment assay, 1 ml of viral concentrate was added to 3 ml *Pseudoalteromonas* sp. QC-44 exponentially growing in liquid Zobell medium (1.0 g yeast extract, 5 g peptone, 250 ml MQ water and 750 ml 30 kDa filtered seawater). After 24h of incubation in the dark, the mixture was centrifuged (5,000 x *g*, 10 min) and the supernatant was filtered through a 0.22 μm filter to remove any remaining bacterial cells. Phage enrichment was confirmed by plaque assay, in which 100 μl phage sample from 10x dilution series was combined with 400 μl of liquid bacterial culture (~10^8^ cells) and plated using the agar overlay technique by adding 3.5 ml of molten soft agar (0.5% agar in Zobell; 50ºC). After plating, a well-resolved plaque was picked from the lawn of host cells and eluted with MSM buffer (450 mM NaCl, 50 mM MgSO_4_ x 7H_2_0, 50 mM Tris base, pH 8). To ensure clonal phage isolates, each isolate was plaque purified three times. After purification, high titer phages stocks were prepared by adding 5 ml of MSM to fully lysed plates. The plates were incubated on a shaker (110 RPM) for 40 min and the phage-MSM solution was transferred to a sterile tube and centrifuged at 5,000 x *g* for 10 min where after the supernatants were 0.22 μm filtered and stored at 4ºC in the dark.

### CsCl purification

Phages for transmission electron microscopy and virion structural proteome analysis were further purified by CsCl centrifugation [57]. Briefly, phage lysate from ~20 fully lysed plates was concentrated using polyethylene glycol (PEG). Here, 3.25 g NaCl was added to 50 ml of filtered phage lysate. The mixture was incubated 1 h at 4ºC in the dark followed by centrifugation at 11,000 x *g*, 10 min. The pellet was discarded and PEG 8000 (10%) was added to the supernatant. After an incubation of 1 h at 4ºC in the dark, it was centrifuged (10,000 x *g*, 10 min). The supernatant was discarded and the pellet was resuspended with MSM buffer. The centrifuge tube (Ultra-Clear, Beckman, Fullerton, CA, USA) was layered with 1.125 ml each of (1) 1.7 g CsCl ml^-1^, (2) 1.5g CsCl ml^-1^, (3) 1.45 CsCl ml^-1^ and (4) 1.2 g CsCl ml^-1^, and finally topped with the viral concentrate and centrifuged (102,000 x *g*, 4 h). A turbid white line containing the phages was removed with a syringe (2 ml total volume) and dialyzed (Slide-A-Lyzer Dyalisis Cassete G2 10 K MWCO, Rockford, IL, USA) three times in 1 liter buffer for at least 1 hour, (1 M Tris-HCl pH 8, 10 mM MgCl_2_) containing three sequentially decreasing NaCl concentrations at each buffer change (3 M NaCl; 1.8 M NaCl; 0.6 M NaCl).

### Electron microscopy of *Pseudoalteromonas* phage B8b

Transmission electron microscopy grids were prepared by placing 10 µl of CsCl-purified lysate (see above) onto 200 mesh formvar-coated copper grids (Ted Pella) for 5 min. The solution was subsequently removed with filter paper and grids were negatively stained with 2% uranyl acetate solution by rinsing the grids with 2 drops of the solution and staining for 45 s with a third drop. The grids were examined using a Philips CM12 microscope with an accelerating voltage of 80 kV. Viral capsid diameter and tail length were determined based on an average of several images and they were measured using ImageJ software (US National Institutes of Health, Bethesda, MD, USA; [37, 58].

### One-step growth experiments

The burst sizes and one-step growth curves were determined as described by Weiss et al. [59], with minor modifications. One milliliter of *Pseudoalteromonas* sp. QC44 overnight culture was transferred to 10 ml of fresh 20% Zobell media and incubated with shaking (120 RPM) for about 20 min, until the A_600_ was ~ 0.02 (mid-log phase), which was equivalent to a viable cell count of around 10^8^ cells/ml. The concentration of bacterial cells at A_600_ ~ 0.02 was verified by flow cytometry [60]. One milliliter of the bacterial culture was then transferred to an eppendorf tube and mixed with phage at a multiplicity of infection of 0.1. The mixture was incubated at room temperature for 15 min to allow phage adsorption. After this adsorption, the mixture was diluted to 10^-2^ in 20 ml of 20% Zobell media to prevent further adsorption of phage. Samples were removed to enumerate total and free phage concentration. In order to detect the free phages, samples were 0.22 µm filtrated before plating. The number of phages in both cases was determined, in duplicate, using the double-agar-layer method as described above. Finally, burst size was calculated as described in [61]. Briefly, burst size was measured as the ratio of the final count of liberated phage particles to the initial count of infected bacterial cells during the latent period.

### Phage specificity

To determine phage host range and bacterial susceptibility, a cross infectivity test was done where plaque assays for each virus–bacteria combination were performed with phage B8b on 52 strains of *Pseudoalteromonas* spp. as well as 34 strains of *Alteromonas* spp., *Marinobacter* spp., *Vibrio* spp., Bacteroidetes, *Nereida* spp., and *Erytrobacter* spp. (S1 Table) using 100 μl of two different phage stock dilutions (10^-5^ and 10^-8^). Lysis was evaluated after overnight incubation in the dark. Once the bacterial strains showed phage susceptibility in the first test, a more thorough analysis was performed to determine the efficiency of infection on each strain. Here, plaque assays were performed with a range of 10x diluted phage stock and plaques were enumerated after 1 and 2 days incubation. Efficiency of infection was expressed in relative PFU (Plaque Forming Units), where the highest was set to 100%.

### Pulsed-field gel electrophoresis

Phage genome size was determined by pulsed-field gel electrophoresis (PFGE) [15]. For this procedure phage lysate was concentrated by Amicon Ultra-15 centrifugal filter units (Millipore) from 5 ml to a final volume of 400 µl. Of this, equal amounts (400 µl) were mixed with melted 1.6% low‐melting‐point agarose (Pronadisa), transferred to plugs molds, left to solidify at room temperature for a few minutes and then kept for 15 minutes at 4ºC. Plugs were incubated overnight at 50ºC in ESP (0.5 M EDTA, pH 9, 0.1% N‐laurylsarcosine and 1 mg ml^-1^ proteinase K) and stored at 4ºC until further analysis. PFGE was performed on a CHEF‐DR III system (Bio‐Rad) using 1% agarose gel (LE agarose SeaKem n.50005 BERLABO S.A.). The gel was run for 22h in 0.5X TBE buffer (1X TBE is 89 M Tris, 2 mM EDTA, and 89 mM boric acid, pH 8.3) at a 5.0‐15.0 seconds switch time, 6V cm^-1^ and an included angle of 120 degrees. After electrophoresis, the gel was stained with SYBR Gold (Molecular probes, 10.000X) diluted to 10^-4^ in 150 ml of TBE for 15 min and washed with MQ water for 15 min. Lambda Low Range (New England Biolabs) was used as molecular size marker. We did three replicates of the PFGE and all of them give us the same genome size estimation.

### Viral DNA purification and genome sequencing

Viral DNA was obtained using the Lambda Wizard DNA kit (Promega Corp. Madison, WI) [62, 63]. Phage lysate from ~15 fully lysed plates were concentrated using polyethylene glycol as described earlier (CsCl purification section). One ml of Purification Resin (Promega, product A7181 Madison WI) was added to 1.5 ml of phages (the PEG pellet resuspended with MSM) and mixed gently by inverting the tube. The mixture was loaded onto a mini-column (Promega, product A7211 Madison WI) through a 5 ml syringe attached to the column, pushing the mixture through with the syringe plunger. The column was then washed with 2 ml 80% isopropanol, the syringe was removed and the mini-column placed into a 1.5 ml eppendorf tube and centrifuged (10,000 x g, 2 min, room temperature) to remove any remaining liquid. Phage DNA was eluted from the column by adding 100 ml TE buffer (80ºC), and the DNA was recovered in a 1.5 ml eppendorf tube through centrifugation (10,000 x g, 30 s, room temperature). Phage DNA was stored at-20ºC. The genome was sequenced by the Life sequencing company (Valencia, Spain) using the standard shotgun sequencing reagents and a 454 GS FLX Titanium Sequencing System (Roche), according to the manufacturer’s instructions.

### Genome assembly and annotation

B8b phage genome sequences were assembled into 4 contigs using Newbler (Roche). In the absence of complete genome coverage, attempts were made to close the gaps using PCR and by direct Sanger sequencing. Forward and reverse primers were designed for every contig using PRIMER3 VERSION 0.4.0 [64], producing a 300–400 bp overlap among the different contigs (see S2 Table). Unfortunately, we failed to close the genome since we could not get PCR products derived from any primer and contig combination. Moreover, we did not obtain any good enough sequence from direct sequencing using any of the designed primers.

ORFs were predicted using a pseudo-automated pipeline where the ORFs were assigned by GeneMark Heuristic [65] followed by refinement through synteny and maximizing ORF size where alternative start sites were present. Gene identification and annotation was done using the BLASTP program against the NCBI non-redundant (nr) database (e-value cut off <0.001, August 2013).

Accession number of the B8b phage genes was deposited into NCBI under the following accession numbers: KJ944830, KM000061, KM000062 and KM000063.

### Proteome analysis

Phages were harvested with MSM from fully lysed plates and CsCl purified as described above. The purified phage particles were prepared prior to 2d-LC-MS/MS analyses using an optimization of the FASP kit (Protein Discovery, Knoxville, TN) [66]. All reagents were provided for in the kit. Briefly, purified phages were re-suspended in 8M Urea/10mM DTT, denatured and passed over the 30kDa filter, then washed with 8M Urea and treated with Iodoacetamide (IAM) to label cysteine residues. IAA was washed away with 8M Urea and then 50mM Ammonium Biocarbonate. Sequencing grade trypsin was then added and digestion processed overnight. The next day peptides were eluted from the 30kDa filter via Ammonium Biocarbonate buffer, NaCl buffer and water/0.1% Formic acid. Three aliquots were prepared per sample and frozen at-80C until 2d-LC-MS/MS analyses. The FASP prepared peptides (>500 ng) were loaded onto the back column of a split phase 2D column (~3–5cmSCX and 3–5cm C-18) (all packing materials purchased from Phenomenex, Torrance, CA). The column was loaded to the HPLC and washed with 100% aqueous solution for 5 min, followed by a ramp from 100% aqueous to 100% organic solution for 10 min. The column [66] was connected to a front column (RP C-18, 15cm) with a nanospray source on LTQVelos and run for 5–12 h two dimensional separation of increasing salt pulses (ammonium acetate) followed by water to organic gradients (see [66]. All instrument were run in a data-dependent manner as previous described [67, 68]. To recruit peptides to the phage genomes, the resulting MS/MS spectra were searched against a database consisting of annotated phage proteins, all phage ORFs > 30 amino acids (aa) (to identify ORFs possibly missed through the annotation), and proteins from sequenced *Pseudoalteromonas* bacteria (*Pseudoalteromonas haloplanktis* TAC125, *Pseudoalteromonas* sp. TW-7, *Pseudoalteromonas atlantica* T6c, *Pseudoalteromonas tunicata* D2) and eukaryotic organisms (human and mouse) to use as indicator for false positives. Data analyses were performed using SEQUEST and filtered with DTA Select with conservative filters [67]. For proteomics, databases, peptide and protein results, MS/MS spectra and supplementary tables are archived and available at https://maple.lsd.ornl.gov/mspipeline/sullivan/, while MS.raw files or other extracted formats are available upon request.

### Phylogenetic analysis

DNA polymerase, phage portal protein, and phage large terminase amino acid sequences of known bacteriophages (S3, S4, S5 Tables) were used to investigate the phage B8b phylogeny. Multiple sequence alignment was automatically performed using the program ClustalW (default parameters) [69]. Maximum likelihood trees were built using the JTT model [70] with bootstrap analysis (1000 replicates) using MEGA version 5.1 [71].

### Fragment recruitment analysis of B8b phage on Pacific Ocean Viral metagenomics (POV)

Fragment recruitment analyses (FRA) were performed to get a sense of the phage B8b relative abundance in the 32 marine viral metagenomes from the Pacific Ocean Virome [26] (available at CAMERA (http://camera.calit2.net) under the following project accessions: CAM_P_0000914 and CAM_P_0000915). We used the Reciprocal Best Blast approach (RBB) [72] applying the same rationale to that employed elsewhere [43]. Briefly, individual metagenomic samples are made into a BLAST database, and then the predicted ORFs of the phage B8b are searched against it using TBLASTn. After this initial blast, hits to the POV database are extracted and become the query for a second BLAST search (BLASTx) against an internal protein genome reference database with a total size of 8,512,217 ORFs that included: (i) protein viral genomes (Refseq Release 60; 4958 genomes and 163,830 ORFs), (ii) bacterial genomes (RefSeq Release 60; 197,527 contigs and 8,348,231 ORFs) and (iii) the *Pseudoalteromonas* phage B8b (4 Contigs, 55 ORFs). Only those metagenomic sequences that returned as a best hit a sequence from the genome of the *Pseudoalteromonas* phage B8b were extracted from the database and counted as hits for subsequent step. Finally, to calculate the relative abundance of B8b phage and two other phages used as reference genomes (the abundant Pelagiphage HTV0C10P (KC465898) and the non marine Enterobacteriaphage T4 (NC_000866)) in the POV dataset, we normalized the number of hits to: 1) protein length, 2) sequencing depth and 3) mean abundances across the 32 POV metagenomes. This was calculated by dividing the number of hits (H) by the total number of sequences (N) and the amino acid length of the hit protein (L). Finally, to avoid large numbers of significant figures, the abundances were rescaled to the mean abundances (mean normalization) across all samples where the numerator is calculated from individual samples and the denominator is calculated from all the samples.

$$A_{pep}\text{ = }\frac{\left(H\times N^{-1}\times L^{-1}\right)}{\left(H\times N^{-1}\times L^{-1}\right)}$$

## Results and Discussion

### Morphology and biology characterization of *Pseudoalteromonas* phage B8b

**Figure 1:**
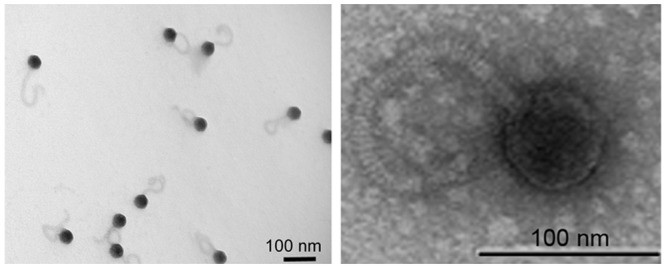
Transmission electron micrograph showing negatively stained *Pseudoalteromonas* phage B8b.

Phage B8b was isolated from Blanes Bay Microbial Observatory (BBMO), an oligotrophic surface coastal site in the NW Mediterranean Sea, and it formed clear, round plaques when grown on its host of isolation, *Pseudoalteromonas* sp. QC-44. Morphological examination showed that phage B8b belonged to the *Siphoviridae* family based on ICTV rules of nomenclature [73] and had an isometric capsid of 49.8 ±1.6 nm in diameter connected to a long and flexible tail of 175.5 ±3.2 nm in length (Fig. 1).

**Figure 2:**
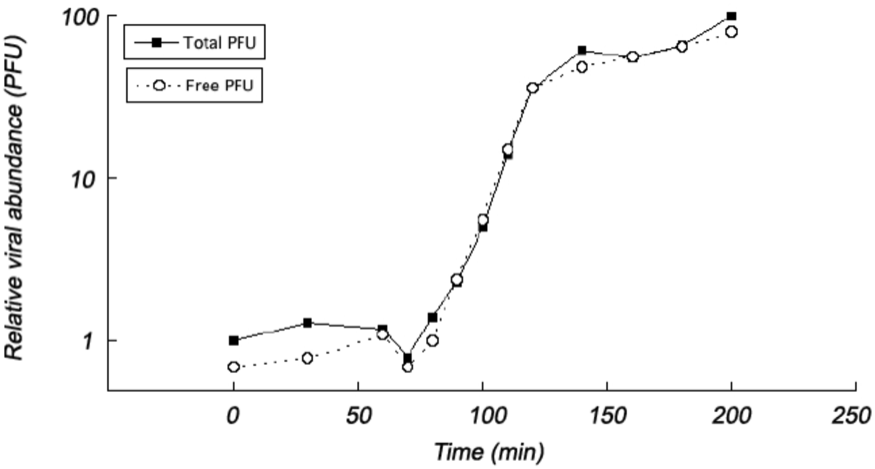
One-step growth curve of *Pseudoalteromonas* phage B8b on *Pseudoalteromonas* sp. QC-44 strain (▮, total PFU; Ο free PFU).

The one-step growth curve of phage B8b showed a latent period of 70 min and approximately 172 new viral particles were released from each infected *Pseudoalteromonas* sp. QC-44 cell (Fig. 2). These values differed from the marine *Pseudoalteromonas* phage PM2, which produced 300 viral particles per infected cell about 70–90 min after infection [74], as well as other marine siphoviruses, e.g. *Vibrio* phage SIO-2, which had a latent period of 45–60 min and an average burst size of 60 [75] or the cyanosiphovirus S-BBS1, which had a 540 min (9 h) of latent period and approximately 250 progeny viruses were produced per infected host cell [76]. However, this is not surprising as burst size and latent period is known to vary between phages, but also depend on which host they infect [61], nutrient availability, specific growth rate of the host, and temperature [77]. Additionally, marine bacteria thrive under lower nutrient concentrations than provided in the lab and, consequently, *in situ* burst size is likely smaller than the values we measured [77].

**Figure 3:**
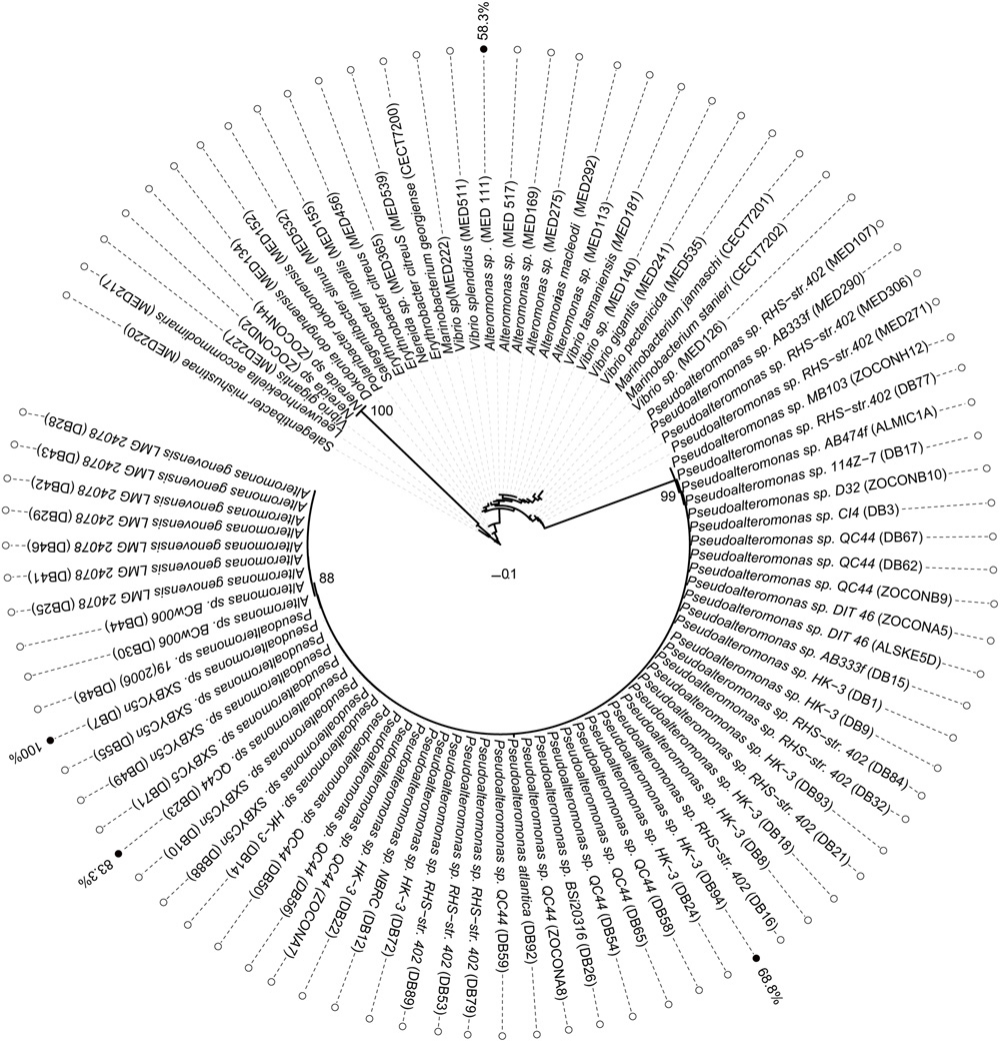
Phylogenetic analyses of the bacterial hosts used to test the *Pseudoalteromonas* phage B8b phage host range. Bacterial strains infected by siphovirus B8b are labeled in black and the efficiency of phage B8b in hosts infected is indicated. Efficiency is expressed in relative PFU, where the highest was set to 100% and the same phage titer dilution was used for all the bacterial strains (10^6^). Names in brackets are strain designations (See S1 Table for more information).

To examine the host range of the isolated phage, infectivity was tested on 52 *Pseudoalteromonas* sp., 15 *Alteromonas* sp., 8 *Vibrio* sp. strains, 3 *Marinobacterium*, sp., all those belonging to Gammaproteobacteria class plus 5 Bacteroidetes (Flavobacteria) and 6 Alphaproteobacteria (Rhodobacterales and Sphingomonadales) (S1 Table). All tested bacterial strains were isolated from the same BBMO marine station as the phage. Phage B8b only infected 3 of 52 *Pseudoalteromonas* spp. strains (Fig. 3) and the phage’s efficiency of infection range between 67–100% on the 3 different *Pseudoalteromonas* strains (Fig. 3). These narrow host range findings agree with previous *Pseudoalteromonas* phage host ranges—PM2 infected 2 of 13 *Pseudoalteomonas* strains [74], H105/5 infected 3 of 52 *Pseudoalteromonas* strains [50], and RIO-1 infected 4 of 11 *Pseudoalteomonas* strains [55]. The use of a single-host enrichment method in this study might bias the results towards a narrow host range phage [78]. However, this narrow siphovirus host range is consistent with previous findings on *Pseudoalteromonas* phages and cyanophages [50, 79] and compared with extended myovirus host range, but contrasts observations in the *Cellulophaga* phages [13, 80].

Of the 37 non-*Pseudoalteromonas* bacterial strains tested, phage B8b was only able to infect a single *Alteromonas* strain. This strain belongs to a different family (Alteromonadaceae) than the phage B8b original host (Pseudoalteromonadaceae) with only 86% nucleotide identity at the 16S rRNA locus between the two bacterial strains (Fig. 3). Also, a lower efficiency of infection (58%) was observed on *Alteromonas* sp. compared to the infection on the host of isolation (Fig. 3). Previously, phages infecting across genera boundaries have been reported, but this is commonly among large myoviruses, like cyanophages infecting *Prochlorococcus* [79], enterophage LG1 and AR1 [81], and vibriophage KVP40 [82], and the two genera do not represent different families of host microbes. Among siphoviruses, one isolate has been reported to infect two bacterial strains of different genera in wastewater [83, 84], but no such cross-genera infections have been reported for siphoviruses from the marine environment. The fact that this phage may infect over genus boundaries highlights the potential of this phage for mediating transduction, and thereby increasing microdiversity, not only among closely related bacterial strains but also across larger taxonomic space [85].

### Structure and general properties of the siphovirus B8 genome

**Figure 4:**
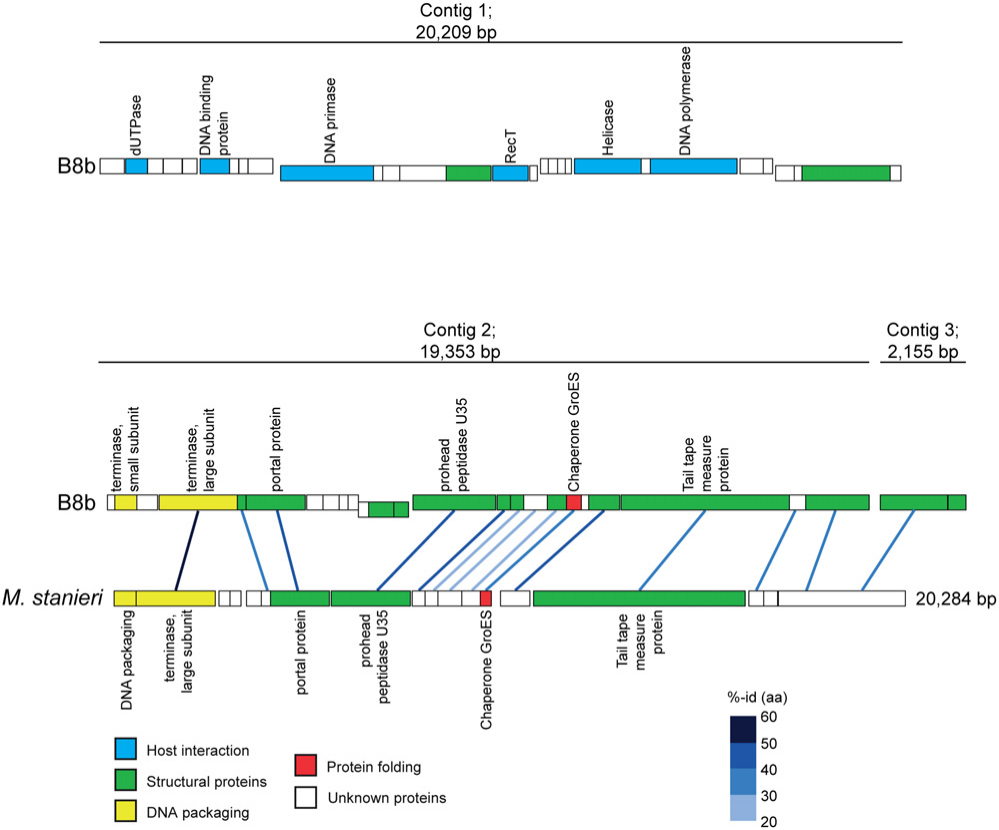
Genome structure of *Pseudoalteromonas* phage B8b represented by 3 contigs and genome comparison with the putative prophage of *Marinobacterium stanieri*. Lines drawn between the genomes represent shared sequence similarity, which is given next to each line as percentage amino acid (aa) identity (id).

While the PFGE analyses predicted that *Pseudoalteromonas* phage B8b had a genome size of 46 kb (S1 Fig.), the combined length of the 4 sequenced contigs was 42,700 bp. These represented two major contigs (20,209 and 19,353 bp) and two short contigs (2,155 and 1,012 bp). Although these contigs could not be closed, probably due to existence of host DNA in the phage DNA sample, the PFGE sizing as compared to the summed contig lengths suggests that about 90% of the phage genome was sequenced. Moreover, the obtained banding pattern in the PFGE gel (S1 Fig.) suggests that the phage had a concatemeric genome where multiple copies of the original DNA were linked in a continuous series of multiples of the predicted genome size. This can be produced by rolling circle replication and/or recombination and is a common replication strategy for dsDNA bacteriophages genomes [86]. The 4 genomic contigs had an average G+C content of 50% and contained 58 predicted open reading frames (ORFs; Table 1). Thirty of these ORFs had significant sequence similarity to proteins in GenBank, but only 12 could be annotated to a function (Table 1), which is similar to other previously sequenced marine *Pseudoalteromonas* phages [49, 54, 55] and siphoviruses [75, 87–89]. Among the genes with detected similarity, 40% were most similar to viruses, 27% to prophages and 33% showed similarity to genes detected in bacterial genomes (Table 1). *Pseudoalteromonas* phage B8b displayed two distinctive functional modules (Fig. 4). A replication module was found in contig 1, which had several genes bioinformatically identified as involved in DNA replication and nucleotide metabolism, such as DNA primase (Contig1_ORF10), helicase (Contig1_ORF21) and DNA polymerase (Contig1_ORF23). Furthermore, the majority of ORFs with the highest similarity to phages (9 of 12) were detected in contig 1, being 7 of them most similar to siphoviruses (Table 1). A packaging/structural module was observed in contig 2 and contained genes that encoded proteins including phage terminases (Contig2_ORF2 and ORF4), phage portal protein (Contig2_ORF6), prohead peptidase (Contig2_ORF14), and tail tape measure protein (Contig2_ORF22). No genes involved for transcription regulatory functions were identified.

**Table 1:** Genomic annotation of *Pseudoalteromonas* siphovirus B8b and structural proteomic results.

	**Genomic data**									**Proteomic data**		
**Contig_ORF**	**Nucleotide start position**	**Nucleotide end position**	**Strand**	**Product lenght (aa)**	**% aa ID**	**Predicted identity or function of product**	**Strain with closest hit (Evalue)**	**Accesion number**	**Taxonomy**	**Sequence count**	**Spectral count**	**Sequence coverage (%)**
Contig1_ORF1	7	630	+	208		Hypothetical phage protein	Non-significant					
Contig1_ORF2	676	1221	+	182	42.0	dUTPase	*Lactococcus* phage Q33 (8.0E-13)	AFV51054.1	*Siphoviridae*, Caudovirales			
Contig1_ORF3	1218	1625	+	136		Hypothetical phage protein	Non-significant					
Contig1_ORF4	1622	2128	+	169		Hypothetical phage protein	Non-significant					
Contig1_ORF5	2121	2456	+	112		Hypothetical phage protein	Non-significant					
Contig1_ORF6	2536	3297	+	254	37.0	DNA binding protein	*Salmonella* phage E1 (4.0E-45)	WP_003849806.1	*Siphoviridae*, Caudovirales			
Contig1_ORF7	3282	3500	+	73		Hypothetical phage protein	Non-significant					
Contig1_ORF8	3503	3760	+	86		Hypothetical phage protein	Non-significant					
Contig1_ORF9	3753	4376	+	208		Hypothetical phage protein	Non-significant					
Contig1_ORF10	6914	4560	-	785	35.0	DNA primase	*Salmonella* phage FSL SP-062 (1.0E-72)	AGF89287.1	*Siphoviridae*, Caudovirales			
Contig1_ORF11	7155	6919	-	79		Hypothetical phage protein	Non-significant					
Contig1_ORF12	7580	7152	-	143	47.0	Conserved hyphotetical phage protein	*Edwardsiella* phage MSW-3 (6.0E-101)	YP_007348969	*Myoviridae*, Caudovirales			
Contig1_ORF13	8746	7580	-	389		Hypothetical phage protein	Non-significant					
Contig1_ORF14	9873	8749	-	375	33.0	Conserved hyphotetical phage protein	*Salmonella* phage FSL SP-062 (2.0E-33)	AGF89282.1	*Siphoviridae*, Caudovirales	3	8	10.9
Contig1_ORF15	10783	9878	-	302	42.0	RecT protein	*Marichromatium purpuratum* 984 (1.0E-4)	WP_005220619	Gammaproteobacteria, Chromatiales			
Contig1_ORF16	11037	10813	-	75	51.0	Conserved hyphotetical phage protein	*Acinetobacter* phage Ac42 (2.0E-20)	YP_004009376	*Myoviridae*, Caudovirales			
Contig1_ORF17	11117	11281	+	55		Hypothetical phage protein	Non-significant					
Contig1_ORF18	11286	11549	+	88		Hypothetical phage protein	Non-significant					
Contig1_ORF19	11546	11743	+	66		Hypothetical phage protein	Non-significant					
Contig1_ORF20	11709	11891	+	61		Hypothetical phage protein	Non-significant					
Contig1_ORF21	11958	13628	+	557	35.0	Helicase	*Salmonella* phage FSL SP-062 (3.0E-92)	AGF89284.1	*Siphoviridae*, Caudovirales			
Contig1_ORF22	13621	13857	+	79		Hypothetical phage protein	Non-significant					
Contig1_ORF23	13847	16057	+	737	39.0	DNA polymerase	*Salmonella* phage FSL SP-062 (9.0E-145)	AGF89344.1	*Siphoviridae*, Caudovirales	2	3	3.9
Contig1_ORF24	16103	16705	+	201		Hypothetical phage protein	Non-significant					
Contig1_ORF25	16705	16938	+	78		Hypothetical phage protein	Non-significant					
Contig1_ORF26	17471	17001	-	157	40.0	Conserved hyphotetical phage protein	*Klebsiella* phage phiKO2 (2.0E-17)	YP_006634.1	*Siphoviridae*, Caudovirales			
Contig1_ORF27	17695	17468	-	76	41.0	Conserved Hypothetical phage protein	*Alishewanella jeotgali* KCTC 22429 (7.0E-11)	WP_008951684	Gammaproteobacteria, Alteromonadales			
Contig1_ORF28	19877	17688	-	730	30.0	Conserved Hypothetical phage protein	*Marinobacterium stanieri* S30 (5.0E-28)	WP_010325175	Gammaproteobacteria, Alteromonadales (Prophage)	4	4	7.8
Contig1_ORF29	20170	19874	-	99	33.0	Conserved Hypothetical phage protein	*Pseudoalteromonas* sp. BSi20652 (7.0E-5)	WP_008172253	Gammaproteobacteria, Alteromonadales			
Contig2_ORF1	256	486	+	77		Hypothetical phage protein	Non-significant					
Contig2_ORF2	410	1006	+	199	25.0	Small terminase subunit	*Escherichia* phage vB_EcoM_ECO1230–10 (7.12E-7)	ADE87936.1	*Myoviridae*, Caudovirales			
Contig2_ORF3	990	1502	+	171	28.0	Conserved hyphotetical phage protein	*Shewanella frigidimarina* NCIMB 400 (8.32E-4)	YP_750332	Gammaproteobacteria, Alteromonadales			
Contig2_ORF4	1519	3510	+	664	59.0	Phage large terminase subunit	*Marinobacterium stanieri* S30 (3.00E-125)	WP_010322164	Gammaproteobacteria, Alteromonadales (Prophage)			
Contig2_ORF5	3514	3729	+	72		Hypothetical phage protein	Non-significant			5	10	76.40
Contig2_ORF6	3719	5218	+	500	40.0	Phage portal protein	*Marinobacterium stanieri* S30(5.0E-126)	WP_010322159	Gammaproteobacteria, Alteromonadales (Prophage)	18	46	42.20
Contig2_ORF7	5232	5660	+	143		Hypothetical phage protein	Non-significant					
Contig2_ORF8	5662	6108	+	149		Hypothetical phage protein	Non-significant					
Contig2_ORF9	6053	6283	+	77		Hypothetical phage protein	Non-significant					
Contig2_ORF10	6264	6524	+	87		Hypothetical phage protein	Non-significant					
Contig2_ORF11	6821	6525	-	99	31.0	Conserved hyphotetical phage protein	*Pseudoalteromonas* phage RIO-1 (2.0E-6)	YP_008051111.1	*Podoviridae*, Caudovirales			
Contig2_ORF12	7423	6803	-	207	40.0	Conserved Hypothetical phage protein	*Pseudoalteromonas* phage RIO-1 (2.0E-6)	YP_008051111.1	*Podoviridae*, Caudovirales	6	10	37.30
Contig2_ORF13	7800	7423	-	126		Hypothetical phage protein	Non-significant			6	22	57.90
Contig2_ORF14	7912	9966	+	685	44.0	Peptidase U35 phage prohead HK97	*Marinobacterium stanieri* S30 (3.0E-168)	WP_010322158	Gammaproteobacteria, Alteromonadales (Prophage)	28	937	42.70
Contig2_ORF15	10022	10360	+	113	48.0	Conserved hyphotetical phage protein	*Marinobacterium stanieri* S30 (1.0E-17)	WP_010322157	Gammaproteobacteria, Alteromonadales (Prophage)	7	67	43.00
Contig2_ORF16	10341	10682	+	114		Hypothetical phage protein	Non-significant			3	3	33.60
Contig2_ORF17	10675	11298	+	208	41.0	Conserved hyphotetical phage protein	*Vibrio crassostreae* (3.0E-44)	WP_017059000	Gammaproteobacteria, Vibrionales			
Contig2_ORF18	11295	11771	+	159	30.0	Conserved Hypothetical phage protein	*Marinobacterium stanieri* S30 (4.0E-4)	WP_010322154	Gammaproteobacteria, Alteromonadales (Prophage)	3	3	24.40
Contig2_ORF19	11774	12148	+	125	35.0	Chaperone GroES	*Pseudoalteromonas tunicata* (1.0E-4)	WP_009840504	Gammaproteobacteria, Alteromonadales			
Contig2_ORF20	12148	12318	+	57		Hypothetical phage protein	Non-significant					
Contig2_ORF21	12318	13079	+	254	40.0	Conserved hyphotetical phage protein	*Marinobacterium stanieri* S30 (8.01E-39)	WP_010322152	Gammaproteobacteria, Alteromonadales (Prophage)	9	290	54.90
Contig2_ORF22	13148	17356	+	1403	32.0	Phage tail tape measure protein TP901, core region	*Marinobacterium stanieri* S30 (1.29E-161)	WP_010322151	Gammaproteobacteria, Alteromonadales (Prophage)	46	74	43.50
Contig2_ORF23	17359	17769	+	137	39.0	Conserved hyphotetical phage protein	*Pseudoaltermonas* sp. S9 (8.0E-17)	WP_010490777	Gammaproteobacteria, Alteromonadales			
Contig2_ORF24	17769	19343	+	525	35.0	Conserved Hypothetical phage protein	*Pseudomonas aeruginosa* (7.0 E-73)	WP_019396974.1	Gammaproteobacteria, Pseudomonadales	12	21	36.19
Contig3_ORF1	9	1694	+	562	38.0	Conserved hyphotetical phage protein	*Pseudomonas aeruginosa (5.0 E-82)*	WP_019396974.1	Gammaproteobacteria, Pseudomonadales	12	38	27.63
Contig3_ORF2	1694	2154	+	153		Hypothetical phage protein	Non-significant			3	3	30.10
Contig4_ORF1	12	350	+	113	29.0	Conserved hyphotetical phage protein	*Klebsiella oxytoca* (9.0E-5)	WP_004131755	Gammaproteobacteria, Enterobacteriales			
Contig4_ORF2	343	609	+	89		Hypothetical phage protein	Non-significant					
Contig4_ORF3	824	699	-	42		Hypothetical phage protein	Non-significant					

### Proteomic analysis

Given that only 5 viral structural proteins were identified by sequence similarity, we performed virion structural proteomic analyses to experimentally confirm identified structural genes and determine the remaining unknown structural proteins. The portal protein, prohead peptidase, tail tape measure protein as well as 8 ORFs of unknown function in contig 2 were detected as part of the phage particle (Table 1). Further, the 2 ORFs of unknown function in contig 3 are part of the phage structural particle, as well as two proteins of unknown function in contig 1. Three spectra also matched against the DNA polymerase gene, however, they were considered false positives as the total peptides detected covered <4% of the gene.

### Phylogenetic relationships

In order to get insights into the phylogenetic relatedness of phage B8b compared to other phages, three relevant genes were compared phylogenetically to similar genes: the B8b DNA polymerase, the phage large terminase, and phage portal protein (S2, S3, S4 Figs. and S3, S4, S5 Tables).

DNA polymerase genes are crucial in genomic replication and mutagenic repair and it has been used to define phylogenetic relationships for novel isolated phages [75, 90, 91]. Surprisingly, the B8b DNA polymerase clustered together with several myoviruses (S2 Fig.). Two of them were isolated from marine bacteria (*Edwardsiella* phage MSW-3 and *Klebsiella* phage JDOO1) [92, 93] and most of them were lytic phages, except for *Vibrio* phage CP-T1 that is known to be temperate (i.e., capable of forming a lysogen; [94]). Although DNA polymerases have been suggested to be good phylogenetic markers for investigating viral phylogeny since they offer the greatest number of viral homologs [95], our results showed that this gene’s phylogeny may be incongruent with electron microscopy- and genome-based taxonomy.

The phage large terminase and phage portal proteins are commonly highly conserved among phage genes, possibly due to their specific enzymatic functions [96], and phage phylogeny has been investigated using these genes in several other studies [89, 94, 96–98]. The phage terminases are DNA packaging enzymes that contain the ATPase activity that powers DNA translocation and most terminases also contain an endonuclease that during DNA packaging cuts concatemeric DNA into genome lengths. Terminases must also recognize viral DNA in a pool that may include host DNA [86, 99]. Phage portal proteins, one the other hand, are structurally associated with the phage capsid and facilitate DNA packaging during head assembly [86]. Phylogenetically, both B8b terminase and portal protein were most closely related to *Stenotrophomonas* phage S1 (S3, S4 Figs.), a temperate siphovirus isolated from sewage [100]. They also clustered together with the putative temperate siphoviruses *Synechococcus* S-CBS1 and S-CBS3 (terminase) [98], as well as several temperate myoviruses, like *Acidithiobacillus* phage AcaML (terminase and portal) [101], *Halomonas* phage phiHAP-1 (portal) [102], and *Vibrio* phage VP882 (portal) [103]. Again, these single marker gene based findings are inconsistent; while some are congruent with the morphological observations, some are not.

Together, these results from three phage gene markers suggest the rampant mosacism posited for siphoviruses [104] may also be true in marine siphoviruses since it has been detected not only in phage B8b but also in other marine siphoviruses [49, 89, 98]. In contrast, other phage groups (e.g., T4-like myoviruses) appear to have clear signals of vertical descent, particularly in their core gene sets as observed in isolates [105, 106] and large-scale analyses of field populations [107].

### Distinctive genes in *Pseudoalteromonas* phage B8b

The B8b genome encoded a RecT protein (Contig1_ORF15), which is involved in homologous recombination of importance to a variety of cellular processes, including the maintenance of genomic integrity [108]. It provides means for repair of DNA double-stranded breaks, which can arise during DNA replication as well as after damage by external factors such as irradiation [109]. As a ssDNA-binding protein, RecT promotes ssDNA annealing, strand transfer, and strand invasion *in vitro* [110]. In *Escherichia coli*, homologous recombination is mediated by bacteriophage RecT protein that permits efficient DNA engineering in various *E. coli* hosts [111]. Although integrase or excisionase genes were not identified in our genome, the RecT gene encoded might facilitate the integration of the phage B8b genome into the bacterial hosts genome, opening up for the potential of phage B8b to act as a prophage.

The presence of chaperone GroES (also called chaperonin 10; Contig2_ORF19) in B8b is unique as it is the first time GroES been reported in a siphovirus, while it has previously been detected in myoviruses and podoviruses [13]. Chaperonins are known to promote the correct folding of newly synthesized polypeptides and to prevent aggregation of proteins denatured under stress [112]. In *E. coli*, the genes that encode for GroES/GroEL chaperonin system were first identified as host factors required for bacteriophage morphogenesis and subsequent work established that the GroES and GroEL proteins were essential for the correct assembly of λ proheads and T5 tails [113, 114]. The presence of this gene in phage B8b might point out that possibly could have a more complex viral capsid or tail structure than other siphoviruses, which requires that it provides its own chaperonin.

### Possibility of lysogenic replication strategy

Phage B8b was isolated as a lytic phage and its lytic nature was confirmed by the one-step growth curve (Fig. 2). However, from our phylogenetic analyses and the presence of the RecT protein, phage B8b was closely related to several phages that are known to be able to perform lysogeny. Also, a large number of phage B8b’s structural proteins had their closest blast hits to proteins from the *Marinobacterium stanieri S30* microbial genome (see Table 1), which begged its comparison (Fig. 4). The synteny between phage B8b and this bacterial contig suggests that a prophage is present in this microbial genome, although it could be a relic or defective prophage, which would represent the closest available genome representative to phage B8b. This highlights the possibility that B8b could be a temperate phage and also, it is possible that lysogenic replication of phage B8b might be detected if different host strains are infected [61], other growth conditions, which might promote lysogeny, are used [115], or under changed phage-host density ratios [116, 117]. Lysogeny would be an attractive lifestyle in oligotrophic marine environment, such as the NW Mediterranean Sea source waters, as lysogeny is a survival strategy during conditions of low host cell encounter rates [118–120]. Moreover, the presence of a prophage may be advantageous for the bacterial host. Hommoimunity protects lysogens from infection by closely related phages [121] and it has been also proposed that marine prophages may contribute to host survival in unfavorable environments through the suppression of unnecessary metabolic activities [122].

### Marine Gammaproteobacteria host genes

Given that cyanophage genomes commonly contain “host genes” (e.g., [10, 63, 123] and recent metagenomic findings that such viral-encoded host genes cover broad metabolic categories [124] including nearly all of central carbon metabolism [24], we wondered whether such Auxiliary Metabolic Genes (AMGs, *sensu* [125]) existed in this new phage B8b genome. Of the 58 phage B8b´s ORFs, 10 were bacterial and 8 prophage related, and all eight ORFs were related to Alteromonadales (Gammaproteobacteria). In fact, *Alteromonas* sp. MED111 was the only non *Pseudoalteromonas* strain that could be infected by phage B8b in the host-range assay. If phages can act as vectors to genetically transfer DNA across bacterial taxa through lateral gene transfer (LGT) [85] one would expect to find host genes within phages that infect similar hosts. Lateral gene transfer has been previously observed in cyanophages [10, 126] and *Pseudoalteromonas* phages [49]. Genetic interaction of phage and bacterial genomes has been predicted to be highly specific such in co-evolutionary models [127] although it is well know that phages can infect hosts from different species and even genera [79]. Emerging phage-bacteria interactions are now being viewed as networks rather than coupled simplistic interactions [128]. The genus-crossing host range detected in phage B8b and the fact that many of the genes found in our B8 genomes were related to a prophage, from a different host specifically to the genus *Marinobacter* spp. within Gammaproteobacteria stressing that possibility of genetic exchange between different host genera.

### Relative abundance of phage B8b in Pacific Ocean Viral metagenomes

**Figure 5:**
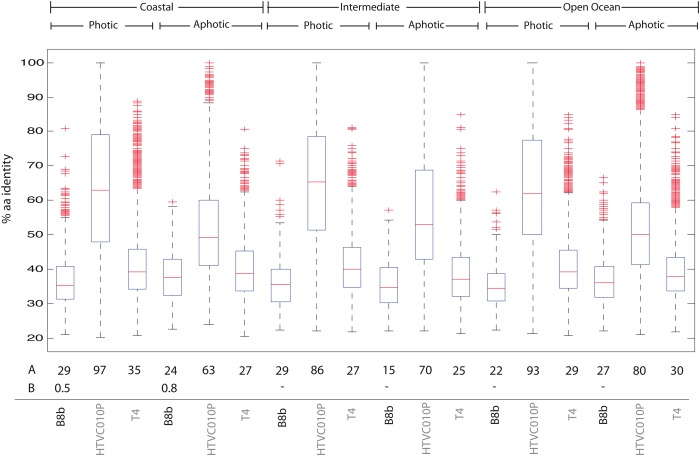
Box plots show the percent amino acid (aa) identity for metagenomics reads (32 metagenomes, POV) recruiting to predicted genes from *Pseudoalteromonas* phage B8b as well as the abundant Pelagiphage HTV0C10P (GenBank No/ KC465898) and the non-marine Enterobacteriaphage T4 (GenBank No. NC_000866). Metagenomes were grouped in 6 categories product of the combination of photic zone and site location as appear in Hurwitz and Sullivan [25]. A) Percentage of the genome that is being covered by the metagenomics reads. B) Percentage of reads that better align to ORFs in the indicated genome (as per bitscore comparison) than the rest of NR. In box plots boxes mark the upper and lower quartile with the median shown in red, whiskers are extended to 1.5 times the interquartile range, finally, red crosses show outliers.

Given the recent availability of a large-scale viral metagenomic dataset (32 Pacific Ocean Viromes, [26]) that was consistently prepared using extensively well-documented quantitative methods [31–36], we wondered whether this relatively novel phage B8b genome was observed in other marine systems and if so how abundant it was. The normalized relative abundance showed that phage B8b was mainly present in the surface, coastal waters with 1.15% assigned reads to phage B8b (Fig. 5). However, an average of 0.46% of the metagenomic reads from deeper, aphotic samples were also recruited to these genomes, which might reflect that a similar host are consistently present through the entire water column since this phage was isolated from surface waters in NW Mediterranean Sea. For environmental phages, these numbers are low when compared to phages for abundant hosts. For example, phages for SAR11, *Synechococcus* and *Prochlorococcus* represented closer to 58.7%, 21.6% and 12.4%, respectively, in diverse ocean viral metagenomes [43]. However, these *Pseudoalteromonas* B8b phage abundances are similar to environmental phages for less abundant hosts—e.g., *Cellulophaga* phages—considered to be representatives of the ‘rare virosphere’ [13]. The percentage of the genome covered by the metagenomics reads in POV database was on average 24.2%, although only 0.65% was exclusive to phage B8b. This suggests that many phage B8b ORFs are likely conserved across a diversity of phages (Fig. 5). Consistent with this hypothesis, the amino acid percentage identity of the predicted proteins for phage B8b genes (24.2%) were similar to that observed for non-marine T4-like phages (29%), but contrasted the identity for pelagiphages (81.4%). Such identities aid in discriminating between whether a new reference genome is itself being observed or is instead the best recruit for reads that likely derive from a more divergent group of phages (see Fig. 5 in [13]). Together we interpret these findings to suggest that this phage is ubiquitous phage in surface, coastal marine waters, but likely another member of the ‘rare virosphere’.

## Conclusions

The *Pseudoalteromonas* phage B8b genome adds a new siphovirus genome for marine Gammaproteobacteria phages. This phage shares many features with available marine siphoviruses and likely represents another member of a ubiquitous class of phages in the ‘rare virosphere’. Its cross-genera host range revealed infection across the genus level and hints of its genome structure suggest that phage B8b may have also a lysogenic lifestyle. Future experimental tests based on phageFISH [129] may allow us to dig into the temperate phage biology within this novel model system.

## Supporting Information

S1 Fig Genome size of phage B8b analyzed by Pulse Field Gel Electrophoresis (PFGE). (TIF)

S2 Fig Phylogenetic relationships of the DNA polymerase across diverse bacteriophages. In green are represented the *Myoviridae* phages, in black the *Siphoviridae* and in blue the *Podoviridae*. Phage B8b is represented in red.
(TIF)

S3 Fig Phylogenetic relationships of the phage large terminase across diverse bacteriophages. In green are represented the *Myoviridae* phages, in black the *Siphoviridae* and in blue the *Podoviridae*. Phage B8b is represented in red.
(TIF)

S4 Fig Phylogenetic relationships of the phage portal protein across diverse bacteriophages. In green are represented the *Myoviridae* phages, in black the *Siphoviridae* and in blue the *Podoviridae*. Phage B8b is represented in red.
(TIF)

S1 Table Bacterial hosts used to test the *Pseudoalteromonas* phage B8b phage host range. Bacterial strain from the phage was isolated is labeled in black. Bacterial strains infected by B8b siphovirus are labeled in red.
(DOCX)

S2 Table Set of designed primers used in the PCR and direct sequencing in order to close the phage B8b genome. (DOCX)

S3 Table Phage DNA polymerase gene sequences used for phylogenetic analysis. (DOCX)

S4 Table Phage large terminase gene sequences used for phylogenetic analysis. (DOCX)

S5 Table Portal protein gene sequences used for phylogenetic analysis. (DOCX)
