# Supplementary figures and images for: Life-Style and Genome Structure of Marine Pseudoalteromonas Siphovirus B8b Isolated from the Northwestern Mediterranean Sea
Source: PLoS One. 2015 Jan 14;10(1):e0114829. doi: 10.1371/journal.pone.0114829 (PMC4294664; doi:10.1371/journal.pone.0114829)

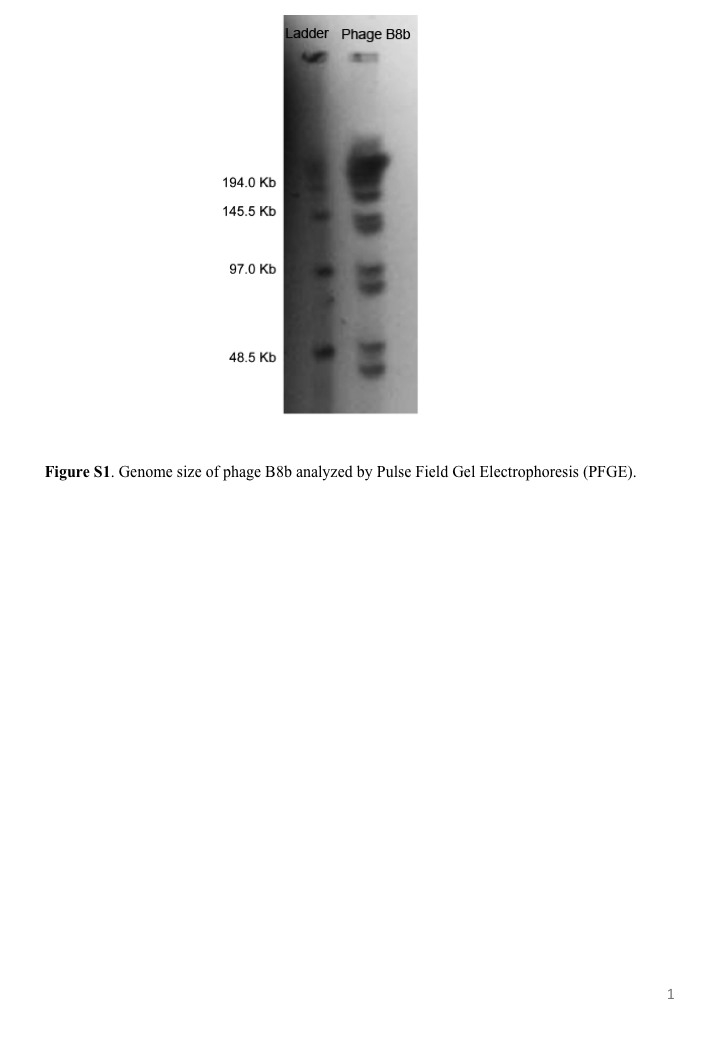

Supplement: S1 Fig — (TIF) [file pone.0114829.s001.tif]

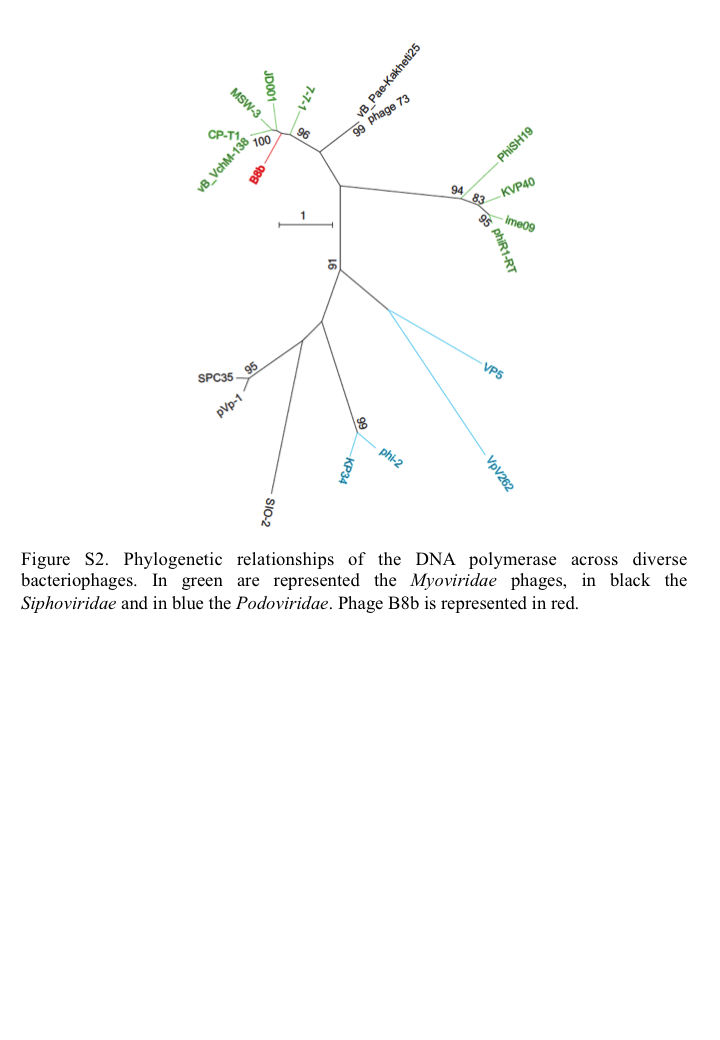

Supplement: S2 Fig — In green are represented the Myoviridae phages, in black the Siphoviridae and in blue the Podoviridae. Phage B8b is represented in red. (TIF) [file pone.0114829.s002.tif]

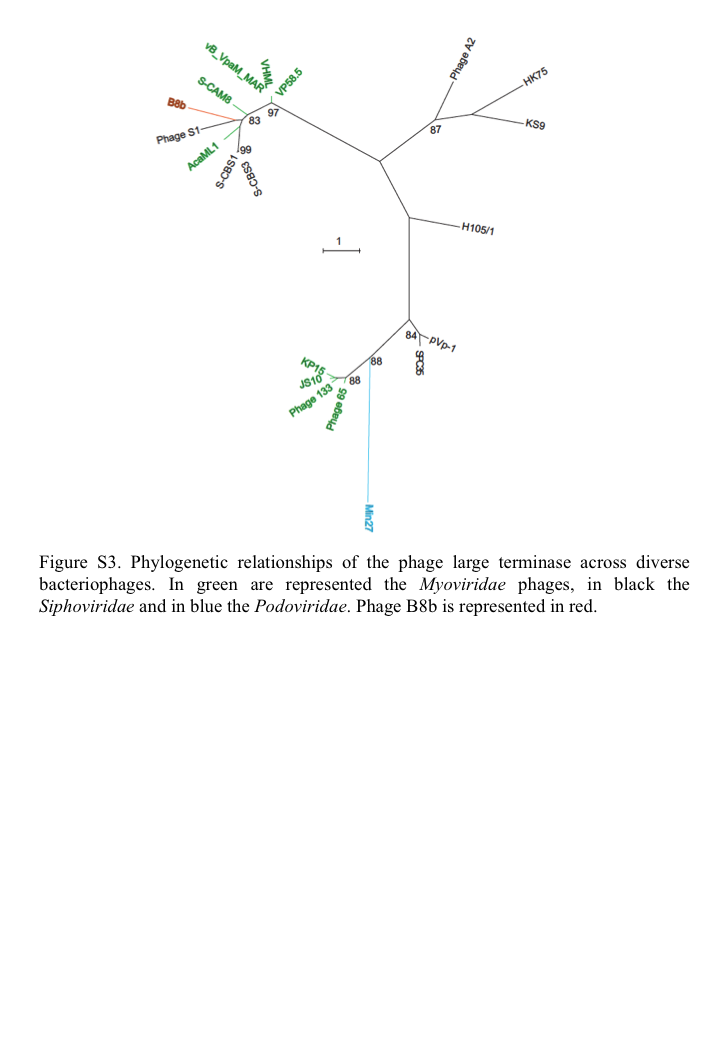

Supplement: S3 Fig — In green are represented the Myoviridae phages, in black the Siphoviridae and in blue the Podoviridae. Phage B8b is represented in red. (TIF) [file pone.0114829.s003.tif]

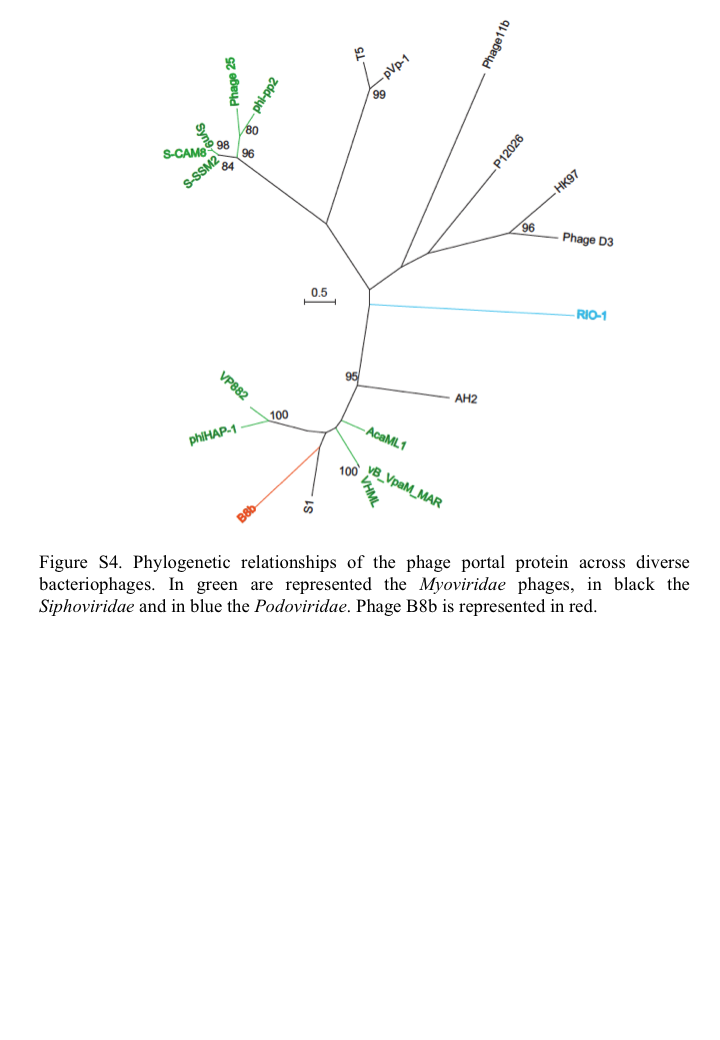

Supplement: S4 Fig — In green are represented the Myoviridae phages, in black the Siphoviridae and in blue the Podoviridae. Phage B8b is represented in red. (TIF) [file pone.0114829.s004.tif]
