# Supplementary material for: Life-Style and Genome Structure of Marine Pseudoalteromonas Siphovirus B8b Isolated from the Northwestern Mediterranean Sea
Source: PLoS One. 2015 Jan 14;10(1):e0114829. doi: 10.1371/journal.pone.0114829 (PMC4294664; doi:10.1371/journal.pone.0114829)
Supplement: S1 Table — Bacterial strain from the phage was isolated is labeled in black. Bacterial strains infected by B8b siphovirus are labeled in red. (DOCX) [file pone.0114829.s005.docx]

**Table S1**. Bacterial hosts used to test the *Pseudoalteromonas* phage B8b phage host range. Bacterial strain from the phage was isolated is labeled in black. Bacterial strains infected by B8b siphovirus are labeled in red.

|  | Taxon | Strain | Isolation year | Isolation location | GenBank Accesion Number |
| --- | --- | --- | --- | --- | --- |
| Gammaproteobacteria/Alteromonadales/Pseudoalteromonadaceae/Pseudoalteromonas | *Pseudoalteromonas* sp. RHS-str.402 | MED306 | 2001 | BBMO | EU253571 |
|  |  | DB89 | 2009 | BBMO | KM609263 |
|  |  | DB32 | 2009 | BBMO | KM609246 |
|  |  | DB77 | 2009 | BBMO | KM609232 |
|  |  | MED271 | 2001 | BBMO | DQ681165 |
|  |  | DB84 | 2009 | BBMO | KM609245 |
|  |  | M107 | 2001 | BBMO | DQ681131 |
|  |  | DB21 | 2009 | BBMO | KM609248 |
|  |  | DB16 | 2009 | BBMO | KM609251 |
|  |  | DB53 | 2009 | BBMO | KM609262 |
|  |  | DB79 | 2009 | BBMO | KM609261 |
|  | ***Pseudoalteromonas* sp. QC44** | **DB23** | **2009** | **BBMO** | **KM609273** |
|  |  | ZOCONA7 | 2009 | BBMO | KM609267 |
|  |  | DB62 | 2009 | BBMO | KM609238 |
|  |  | ZOCONB9 | 2009 | BBMO | KM609239 |
|  |  | ZOCONA8 | 2009 | BBMO | KM609258 |
|  |  | DB50 | 2009 | BBMO | KM609269 |
|  |  | DB54 | 2009 | BBMO | KM609256 |
|  |  | DB56 | 2009 | BBMO | KM609268 |
|  |  | DB67 | 2009 | BBMO | KM609237 |
|  |  | DB59 | 2009 | BBMO | KM609260 |
|  |  | DB58 | 2009 | BBMO | KM609254 |
|  |  | DB65 | 2009 | BBMO | KM609255 |
|  | *Pseudoalteromonas* sp. HK-3 | DB1 | 2009 | BBMO | KM609243 |
|  |  | DB14 | 2009 | BBMO | KM609270 |
|  |  | DB18 | 2009 | BBMO | KM609249 |
|  |  | DB22 | 2009 | BBMO | KM609266 |
|  |  | DB24 | 2009 | BBMO | KM609253 |
|  |  | DB72 | 2009 | BBMO | KM609264 |
|  |  | DB8 | 2009 | BBMO | KM609250 |
|  |  | DB9 | 2009 | BBMO | KM609244 |
|  |  | DB93 | 2009 | BBMO | KM609247 |
|  |  | DB94 | 2009 | BBMO | KM609252 |
|  | *Pseudoalteromonas* sp. SXBYC5n | DB55 | 2009 | BBMO | KM609276 |
|  |  | DB49 | 2009 | BBMO | KM609275 |
|  |  | DB10 | 2009 | BBMO | KM609272 |
|  |  | DB7 | 2009 | BBMO | KM609277 |
|  |  | DB71 | 2009 | BBMO | KM609274 |
|  |  | DB88 | 2009 | BBMO | KM609271 |
|  | *Pseudoalteromonas* sp. AB333f | DB15 | 2009 | BBMO | KM609242 |
|  |  | MED290 | 2001 | BBMO | DQ681167 |
|  | *Pseudoalteromonas* sp. DIT 46 | ALSKE5D | 2009 | BBMO | KM609241 |
|  |  | ZOCONA5 | 2009 | BBMO | KM609240 |
|  | *Pseudoalteromonas atlantica* | DB92 | 2009 | BBMO | KM609259 |
|  | *Pseudoalteromonas* sp. 114Z-7 | DB17 | 2009 | BBMO | KM609234 |
|  | *Pseudoalteromonas* sp. 19(2006) | DB48 | 2009 | BBMO | KM609278 |
|  | *Pseudoalteromonas* sp. AB474f | ALMIC1A | 2009 | BBMO | KM609233 |
|  | *Pseudoalteromonas* sp. BSi20316 | DB26 | 2009 | BBMO | KM609257 |
|  | *Pseudoalteromonas* sp. CI4 | DB3 | 2009 | BBMO | KM609236 |
|  | *Pseudoalteromonas* sp. D32 | ZOCONB10 | 2009 | BBMO | KM609235 |
|  | *Pseudoalteromonas* sp. MB103 | ZOCONH12 | 2009 | BBMO | KM609231 |
|  | *Pseudoalteromonas* sp. NBRC 102015 | DB12 | 2009 | BBMO | KM609265 |
| Gammaproteobacteria/ Alteromonadales/ Alteromonadaceae/Alteromonas | *Alteromonas genovensis* strain : LMG 24078 | DB25 | 2009 | BBMO | KM609281 |
|  |  | DB29 | 2009 | BBMO | KM609284 |
|  |  | DB41 | 2009 | BBMO | KM609282 |
|  |  | DB42 | 2009 | BBMO | KM609285 |
|  |  | DB46 | 2009 | BBMO | KM609283 |
|  | *Alteromonas* sp*.* | MED111 | 2001 | BBMO | DQ681132 |
|  |  | MED113 | 2001 | BBMO | DQ681133 |
|  |  | MED169 | 2001 | BBMO | DQ681147 |
|  |  | MED275 | 2001 | BBMO | DQ681166 |
|  |  | MED517 | 2001 | BBMO | DQ681179 |
|  | *Alteromonas genoviensi* strain I96 | DB28 | 2009 | BBMO | KM609287 |
|  |  | DB43 | 2009 | BBMO | KM609286 |
|  | *Alteromonas* sp. BCw006 | DB30 | 2009 | BBMO | KM609279 |
|  |  | DB44 | 2009 | BBMO | KM609280 |
|  | *Alteromonas macleodii* | M292 | 2001 | BBMO | DQ681168 |
| Gammaproteobacteria/ Vibrionales/ Vibrionaceae/ Vibrio | *Vibrio gigantis* | MED227 | 2001 | BBMO | DQ681158 |
|  | *Vibrio gigantis* | MED241 | 2001 | BBMO | DQ681160 |
|  | *Vibrio pectenicida* | MED535 | 2001 | BBMO | DQ681180 |
|  | *Vibrio* sp. | MED222 | 2001 | BBMO | DQ681157 |
|  | *EnetroVibrio* sp. | MED126 | 2001 | BBMO | DQ681138 |
|  | *Vibrio* sp. | MED140 | 2001 | BBMO | DQ681142 |
|  | *Vibrio splendidus* | MED511 | 2001 | BBMO | EU253592 |
|  | *Vibrio tasmaniensis* | MED181 | 2001 | BBMO | DQ681148 |
| Gammaproteobacteria/ Alteromonadales/ Alteromonadaceae/ Marinobacterium | *Marinobacterium georgiense* | 7200 | 2006 | CECT | NR_114163 |
|  | *Marinobacterium jannaschii* | 7201 | - | CECT | NR_113757 |
|  | *Marinobacterium stanieri* | 7202 | 2006 | CECT | AB021367 |
| Bacteroidetes Bacteroidales/ Bacteroidaceae | *Dokdonia donghaensis* | MED134 | 2001 | BBMO | DQ481462 |
|  | *Polaribacter dokdonensis* | MED152 | 2001 | BBMO | DQ481463 |
|  | *Leeuwenhoekiella accommodimaris* | MED217 | 2001 | BBMO | NR_115725 |
|  | *Salegentibacter mishustinae* | MED220 | 2001 | BBMO | DQ681156 |
|  | *Salegentibacter slinus* | MED532 | 2001 | BBMO | EU253596 |
| Alphaproteobacteria/ Rhodobacterales/ Rhodobacteraceae/ Nereida | *Nereida* sp. | ZOCOND2 | 2009 | BBMO | KM609229 |
|  | *Nereida* sp. | ZOCONH4 | 2009 | BBMO | KM609230 |
|  | *Nereida* sp. | MED365 | 2001 | BBMO | EU253576 |
| Alphaproteobacteria/ Sphingomonadales/ Erythrobacteraceae/ Erythrobacter | *Erythrobacter litoralis* | MED155 | 2001 | BBMO | DQ681145 |
|  | *Erythrobacter citreus* | MED456 | 2001 | BBMO | DQ681172 |
|  | *Erythrobacter citreus* | M539 | 2001 | BBMO | DQ681181 |
| BBMO: Blanes Bay Microbial Observatory (NW Mediterranean Sea) | | |  |  |  |
| CECT: Spanish Type Culture Collection | |  |  |  |  |
