# Supplementary material for: Life-Style and Genome Structure of Marine Pseudoalteromonas Siphovirus B8b Isolated from the Northwestern Mediterranean Sea
Source: PLoS One. 2015 Jan 14;10(1):e0114829. doi: 10.1371/journal.pone.0114829 (PMC4294664; doi:10.1371/journal.pone.0114829)
Supplement: S2 Table — (DOCX) [file pone.0114829.s006.docx]

**Table S2**. Set of designed primers used in the PCR and direct sequencing in order to close the phage B8b genome.

|  | **Set_1** |  | **Set_2** |
| --- | --- | --- | --- |
| **Primer name** | **Sequence (5' to 3')** | **Primer name** | **Sequence (5' to 3')** |
| C1_F1 | ACCCGCTGGTGGCGAGTAAACCCC | F1 | AGCTATCACACCGCCACTAAGCCT |
| C1_1R | ACACCCCTGCCCGTCGGGTAACT | R1 | AGTGCATTTACTGGCGCTGCATCTAGG |
| C2_2R | TCGGGGTGGGGTGTCCGCC | F2 | ACCGTACGGCTGGTAGGGTTGT |
| C3_3R | ACGCACCAGCGCCGTCACGA | R2 | TGCCTTGCGTTACAGGCGCT |
| C4_4R | CCGCATTTCCAACTAGCCGGCGGCATTA | F3 | ACCGCTGAACCTACTGGGGATTTTGA |
|  |  | R3 | TGACGCACCAGCGCCGTCAC |
|  |  | F4 | CCATACGCCACCATACGCCACCA |
|  |  | R4 | TCCAACTAGCCGGCGGCATTACT |
