# Supplementary material for: Life-Style and Genome Structure of Marine Pseudoalteromonas Siphovirus B8b Isolated from the Northwestern Mediterranean Sea
Source: PLoS One. 2015 Jan 14;10(1):e0114829. doi: 10.1371/journal.pone.0114829 (PMC4294664; doi:10.1371/journal.pone.0114829)
Supplement: S3 Table — (DOCX) [file pone.0114829.s007.docx]

**Table S3.** Phage DNA polymerase gene sequences used for phylogenetic analysis.

| **Phage name** | **Family** | **Host** | **Accession** |
| --- | --- | --- | --- |
| Vibrio phage vB_VchM-138 | *Myoviridae* | *Vibrio cholerae* | YP_007006391.1 |
| Vibrio phageCP-T1 | *Myoviridae* | *Vibrio cholerae* ElTor | YP_007003043 |
| Edwardsiella_phage_MSW-3 | *Myoviridae* | *Edwardsiella tarda* | YP_007348961 |
| Klebsiella phage JD001 | *Myoviridae* | *Klebsiella pneumoniae* JDM777 | YP_007392876 |
| Agrobacterium phage 7-7-1 | *Myoviridae* | *Agrobacterium* sp. H13-3 | YP_007006473 |
| Pseudomonas phage vB_Pae-Kakheti25 | *Siphoviridae* | *Pseudomonas aeruginosa* | YP_006299891.1 |
| Pseudomonas_phage_73 | *Siphoviridae* | *Pseudomonas aeruginosa* | YP_001293433.1 |
| Salmonella phage PhiSH19 | *Myoviridae* | *Salmonella Typhimurium* | YP_007008133.1 |
| Vibrio_phage_KVP40 | *Myoviridae* | *Vibrio parahaemolyticus* | NP_899330.1 |
| Enterobacteria phage ime09 | *Myoviridae* | *Escherichia coli* | YP_007004431.1 |
| Yersinia_phage_phiR1-RT | *Myoviridae* | *Yersinia enterocolitica* | YP_007235888.1 |
| Vibrio phage VP5 | *Podoviridae* | *Vibrio cholerae* | YP_024986.1 |
| Vibrio_phage_VpV262 | *Podoviridae* | *Vibrio parahaemolyticus* | NP_640280.1 |
| Pseudomonas_phage_phi-2 | *Podoviridae* | *Pseudomonas fluorescens* SBW25 | YP_003345482.1 |
| Klebsiella phage KP34 | *Podoviridae* | *Klebsiella pneumoniae* | YP_003347671.1 |
| Vibrio phage SIO-2 | *Siphoviridae* | *Vibrio* sp. SWAT-3 | YP_004957553 |
| Vibrio_phage_pVp-1 | *Siphoviridae* | *Vibrio parahaemolyticus* | AFB83871.1 |
| Enterobacteria_phage_SPC35 | *Siphoviridae* | *Salmonella enterica* | YP_004306595.1 |
