# Supplementary material for: Life-Style and Genome Structure of Marine Pseudoalteromonas Siphovirus B8b Isolated from the Northwestern Mediterranean Sea
Source: PLoS One. 2015 Jan 14;10(1):e0114829. doi: 10.1371/journal.pone.0114829 (PMC4294664; doi:10.1371/journal.pone.0114829)
Supplement: S4 Table — (DOCX) [file pone.0114829.s008.docx]

**Table S4.** Phage large terminase gene sequences used for phylogenetic analysis.

| **Phage name** | **Family** | **Host** | **Accession** |
| --- | --- | --- | --- |
| Synechococcus phage S-CBS3 | *Siphoviridae* | *Synechococcus* sp. CB0202 | YP_004421723.1 |
| Synechococcus phage S-CBS1 | *Siphoviridae* | *Synechococcus* sp. CB0201 | YP_004934601.1 |
| Acidithiobacillus phage AcaML1 | *Myoviridae* | *Acidithiobacillus caldus* ATCC 51756 | AFU62879.1 |
| Stenotrophomonas S1 | *Siphoviridae* | *Stenotrophomonas maltophilia* | YP_002321454.1 |
| Synechococcus phage S-CAM8 | *Myoviridae* | *Synechococcus* sp. WH7803 | YP_008125637.1 |
| Vibrio phage vB_VpaM_MAR | *Myoviridae* | *Vibrio parahaemolyticus* | YP_007112478.1 |
| Vibrio phage VHML | *Myoviridae* | *Vibrio harveyi* | NP_758915.1 |
| Vibrio phage VP58.5 | *Myoviridae* | *Vibrio parahaemolyticus* | CAX64983.1 |
| Lactobacillus phage_A2 | *Siphoviridae* | *Lactobacillus casei* | NP_680484.1 |
| Escherichia phage HK75 | *Siphoviridae* | *Escherichia coli* | YP_004934109.1 |
| Burkholderia phage KS9 | *Siphoviridae* | *Burkholderia cepacia* | YP_003090178.1 |
| Pseudoalteromonas phage H105/1 | *Siphoviridae* | *Pseudoalteromonas* sp. H105 | YP_004327112.1 |
| Vibrio phage pVp-1 | *Siphoviridae* | *Vibrio parahaemolyticus* | AFB83996.1 |
| Enterobacteria phage SPC35 | *Siphoviridae* | *Salmonella enterica* | YP_004306624.1 |
| Enterobacteria phage Min27 | *Podoviridae* | *Escherichia coli* O157:H7 str. Min27 | YP_001648943.1 |
| Aeromonas phage 65 | *Myoviridae* | *Aeromonas salmonicida* | YP_004300928.1 |
| Acinetobacter phage 133 | *Myoviridae* | *Acinetobacter johnsonii* | YP_004300751.1 |
| Enterobacteria phage JS10 | *Myoviridae* | *Escherichia coli* | YP_002922510.1 |
