# Supplementary material for: Life-Style and Genome Structure of Marine Pseudoalteromonas Siphovirus B8b Isolated from the Northwestern Mediterranean Sea
Source: PLoS One. 2015 Jan 14;10(1):e0114829. doi: 10.1371/journal.pone.0114829 (PMC4294664; doi:10.1371/journal.pone.0114829)
Supplement: S5 Table — (DOCX) [file pone.0114829.s009.docx]

**Table S5.** Portal protein gene sequences used for phylogenetic analysis.

| **Phage name** | **Family** | **Host** | **Accession** |
| --- | --- | --- | --- |
| Synechococcus phage S-CAM8 | *Myoviridae* | *Synechococcus* sp*.* WH7803 | YP_008125640.1 |
| Synechococcus phage syn9 | *Myoviridae* | *Synechococcus* sp*.* WH 8012 | YP_717798.1 |
| Cyanophage S-SSM2 | *Myoviridae* | *Synechococcus* sp*.* WH8102 | AGH57437.1 |
| Aeromonas phage 25 | *Myoviridae* | *Aeromonas salmonicida* | YP_656382.1 |
| Vibriophage phi-pp2 | *Myoviridae* | *Vibrio parahaemolyticus* | AFN37590.1 |
| Enterobacteria phage T5 | *Siphoviridae* | *Escherichia coli* | YP_006980.1 |
| Vibrio phage pVp-1 | *Siphoviridae* | *Vibrio parahaemolyticus* ATCC 33844 | AFB83998.1 |
| Flavobacterium phage 11b | *Siphoviridae* | *Flavobacterium* sp*.* | YP_112492.1 |
| Marinomonas phage P12026 | *Siphoviridae* | *Marinomonas* sp*.* IMCC12026 | YP_006560242.1 |
| Enterobacteria phage HK97 | *Siphoviridae* | *Escherichia coli* | NP_037699.1 |
| Pseudomonas phage D3 | *Siphoviridae* | *Pseudomonas aeruginosa* | NP_061500.1 |
| Pseudoalteromonas phage RIO-1 | *Podoviridae* | *Pseudoalteromonas marina* | YP_008051121.1 |
| Burkholderia phage AH2 | *Siphoviridae* | *Burkholderia cenocepacia* C6433 | AEY69575.1 |
| Acidithiobacillus phage AcaML1 | *Myoviridae* | *Acidithiobacillus caldus* ATCC 51756 | AFU62881.1 |
| Vibrio phage vB_VpaM_MAR | *Myoviridae* | *Vibrio parahaemolyticus* | YP_007112480.1 |
| Vibrio phage VHML | *Myoviridae* | *Vibrio harveyi* | NP_758916.1 |
| Stenotrophomonas S1 | *Siphoviridae* | *Stenotrophomonas maltophilia* | YP_002321454.1 |
| Halomonas phage phiHAP-1 | *Myoviridae* | *Halomonas aquamarina* | ABY90372.1 |
